# Rescue of Rod Synapses by Induction of Ca_v_ Alpha 1F in the Mature Ca_v_1.4 Knock-Out Mouse Retina

**DOI:** 10.1167/iovs.19-27226

**Published:** 2019-07

**Authors:** Joseph G. Laird, Sarah H. Gardner, Ariel J. Kopel, Vasily Kerov, Amy Lee, Sheila A. Baker

**Affiliations:** 1 Department of Biochemistry, University of Iowa, Iowa City, United States; 2 Molecular Physiology and Biophysics, University of Iowa, Iowa City, United States; 3 Otolaryngology-Head and Neck Surgery, University of Iowa, Iowa City, United States; 4 Department of Neurology, University of Iowa, Iowa City, United States; 5 Iowa Neuroscience Institute, University of Iowa, Iowa City, United States; 6 Ophthalmology and Visual Sciences and the Institute for Vision Research, University of Iowa, Iowa City, United States

**Keywords:** synapse, rod, retina, calcium channel, Cav1.4

## Abstract

**Purpose:**

Ca_v_1.4 is a voltage-gated calcium channel clustered at the presynaptic active zones of photoreceptors. Ca_v_1.4 functions in communication by mediating the Ca^2+^ influx that triggers neurotransmitter release. It also aids in development since rod ribbon synapses do not form in Ca_v_1.4 knock-out mice. Here we used a rescue strategy to investigate the ability of Ca_v_1.4 to trigger synaptogenesis in both immature and mature mouse rods.

**Methods:**

In vivo electroporation was used to transiently express Ca_v_ α_1F_ or tamoxifen-inducible Ca_v_ α_1F_ in a subset of Ca_v_1.4 knock-out mouse rods. Synaptogenesis was assayed using morphologic markers and a vision-guided water maze.

**Results:**

We found that introduction of Ca_v_ α_1F_ to knock-out terminals rescued synaptic development as indicated by PSD-95 expression and elongated ribbons. When expression of Ca_v_ α_1F_ was induced in mature animals, we again found restoration of PSD-95 and elongated ribbons. However, the induced expression of Ca_v_ α_1F_ led to diffuse distribution of Ca_v_ α_1F_ in the terminal instead of being clustered beneath the ribbon. Approximately a quarter of treated animals passed the water maze test, suggesting the rescue of retinal signaling in these mice.

**Conclusions:**

These data confirm that Ca_v_ α_1F_ expression is necessary for rod synaptic terminal development and demonstrate that rescue is robust even in adult animals with late stages of synaptic disease. The degree of rod synaptic plasticity seen here should be sufficient to support future vision-restoring treatments such as gene or cell replacement that will require photoreceptor synaptic rewiring.

Inherited retinal diseases (IRDs) are clinically and genetically diverse.^1^ What unites this group of diseases is the limitations the reduction or loss of vision places on patients' daily activities. Two approaches under heavy investigation for the development of treatments for IRDs are gene therapy and cell replacement. The first FDA-approved gene therapy for IRD, specifically RPE65-associated vision loss,^2^–^5^ has motivated the development of many more gene therapy approaches for treating IRDs, with several currently being tested in clinical trials.^6^–^12^ However, gene therapy will not be the cure for all forms of IRDs; for example, those that cause very rapid and early onset neurodegeneration may not have sufficient living cells remaining by the time a gene therapy vector is available. For situations not amenable to gene therapy, the National Eye Institute is investing in research to develop photoreceptor cell replacement therapies.^13^–^16^ Such studies capitalize on advances in growing photoreceptor progenitor cells from patient-derived iPSC but are not yet ready for testing in humans.

The effectiveness of these potential therapies depends in part on how well repaired or replaced photoreceptors will properly integrate into the existing retinal wiring. Although photoreceptors are terminally differentiated neurons, there is evidence that the synapses can be plastic. This is most often observed in response to some type of stress.^17^ For example, aging results in synaptic retraction and remodeling associated with metabolic stress.^18^–^24^ Mechanical stress results in synaptic injury as seen in retinal detachment or in the progressive IRD, X-linked retinoschisis, due to mutations in *RS1*.^25^–^28^ Synaptic remodeling is also well documented in models of stationary IRDs with alterations in signaling, such as congenital stationary night blindness due to mutations in *CACNA1F* or achromatopsia due to mutations in *CNGA3* or *CNGB*.^29^–^36^ The success of preclinical gene therapies to treat a variety of photoreceptor problems argues that synaptic damage is reversible,^26^,^37^ but the extent to which synapses can reform is unclear. Additionally, if transplantation of healthy photoreceptor precursors into diseased retinas is to be successful, then entirely new synapses will have to form de novo contacts with remodeled horizontal and bipolar cells neurites.^17^,^38^ Investigating mechanisms of photoreceptor synaptogenesis may enhance the development of effective strategies to restore sight.

An integral component of the photoreceptor synapse is Ca_v_1.4. Loss of function for Ca_v_1.4 can result in either a stationary (i.e., CSNB2) or progressive (i.e., CORDX3) IRD.^39^,^40^ Ca_v_1.4 is a voltage-gated Ca^2+^ channel clustered beneath the synaptic ribbon, an organelle that organizes synaptic vesicles to support a high volume of tonic neurotransmitter release.^41^ The influx of Ca^2+^ via Ca_v_1.4 thus provides a voltage-responsive microdomain of Ca^2+^ that is used to trigger fusion of adjacent synaptic vesicles. Additionally, Ca_v_1.4 contributes to synaptic development and maintenance. Ca_v_1.4 is composed of a large pore-forming α_1F_ subunit (encoded by *CACNA1F*) and two accessory subunits, the extracellular α_2_δ-4 (*CACNA2D4*) and intracellular β_2_ (*CACNB2*). Knock out of any subunit in mouse models results in loss of the channel from the synapse and gross morphologic defects of the presynaptic terminal, such as the ribbon failing to elongate and the loss of many functionally related proteins.^31^,^32^,^34^,^42^–^47^ It is not known if these synapses could be triggered to form/regenerate in adult retinas.

In this study, we investigated the regenerative capacity of rod photoreceptor synapses in Ca_v_1.4 knock-out (KO) mice by rescuing Ca_v_ α_1F_ expression in either immature or mature retinas. We found evidence of mature synapse morphology upon Ca_v_ α_1F_ expression, independent of age. Despite limited efficiency in achieving Ca_v_ α_1F_ expression, we also found some animals gained the ability to navigate a visually guided water maze. We conclude that this proof-of-concept rescue study demonstrates that the malformed presynaptic terminal of rods lacking Ca_v_ α_1F_ maintain the potential to regenerate into functional synaptic terminals.

## Methods

### Animals

C57BL/6J (RRID:IMSR_JAX:000664) were used as wildtype (WT) controls, and the Ca_v_1.4 KO mice (RRID:IMSR_JAX:017761) have been previously described.^34^ Mice of both sexes, up to the age of 6 months were used. Mice were housed in a central vivarium, maintained on a standard 12/12-hour light/dark cycle, with food and water provided ad libitum in accordance with the Guide for the Care and Use of Laboratory Animals of the National Institutes of Health. All procedures adhered to the ARVO Statement for the Use of Animals in Ophthalmic and Vision Research and were approved by the University of Iowa IACUC committee.

### Molecular Cloning

All plasmids used in this study are listed in Table 1 and were obtained from Addgene or subcloned using standard PCR-based methods. All inserts were verified by Sanger sequencing (Iowa Institute of Human Genetics, Iowa City, Iowa, USA).

**Table 1:** Plasmids

**Category**	**Insert**	**Full Name**
Ca_v_ α_1F_	FLAG-m*Cacna1F*	pRho-FLAG-mouse Ca_v_ α_1F_, generated in this study by subcloning FLAG-tagged mouse *Cacna1F* in place of Cre in pRho-Cre (Addgene #13779)
i-α_1F_	Inducible FLAG-m*Cacna1F*	pCALNL-α1F, generated in this study by subcloning mouse FLAG-m*Cacna1F* in place of DsRED in pCALNL-DsRED
Tamoxifen-controlled Cre	ER^T2^-Cre-ER^T2^	pCAG-ERT2-Cre-ERT2 (Addgene #13777)
Reporters	GFP	pCAG-mGFP (Addgene #14757)
	mKate2	pRho-mKate2, generated in this study by subcloning mKate2 in place of Cre in pRho-Cre (Addgene #13779)
	DsRED	pCALNL-DsRED (Addgene #13769)
	Inducible SYP-RFP	pCALNL-SYP-RFP, generated in this study by subcloning mouse synaptophysin-mRFP in place of DsRED in CALNL-DsRED

### In Vivo Electroporation

Electroporation was conducted as previously described.^48^–^50^ Briefly, a mixture of 2 to 3 plasmids in sterile PBS (∼4 μg in a volume of ∼0.3 μL) was injected into the subretinal space of one eye of neonatal mice using a 33 G blunt-ended needle. The procedure was performed in the afternoon of the day of birth (postnatal day 0 [P0]). Tweezer-type electrodes placed on the sides of the head were used to deliver transcranial pulses.

### Antibodies and Immunohistochemistry

All antibodies used in this study are listed in Table 2.^32^,^51^ Immunostaining was carried out as previously described.^44^ Briefly, posterior eyecups were collected by dissection, fixed in 4% paraformaldehyde at room temperature for 15 to 20 minutes, cryoprotected in 30% sucrose, and then frozen in OCT (Tissue-Tek; Electron Microscopy Sciences, Hatfield, PA, USA). Radial sections were cut and collected on electrostatically charged glass slides and either labeled immediately or stored at −80°C until use. Blocking buffer consisted of 10% normal goat serum and 0.5% Triton X-100 in PBS. Primary and secondary antibodies (diluted in blocking buffer) were incubated on retinal sections for 1 to 3 hours at room temperature or overnight at 4°C. Images were collected with a 63×, numerical aperture 1.4, oil-immersion objective on either a Zeiss LSM710 confocal (Carl Zeiss, Oberkochen, Germany) or an Olympus FluoView 1000 microscope (Olympus Corp., Tokyo, Japan).

**Table 2:** Antibodies Used for Immunohistochemistry

	**IgG**	**Source**	**Cat No.**	**Concentration**	**RRID**
Ca_v_1.4 no. 168	Rabbit	*Liu et al.^32^	n/a	1:500	n/a
RIBEYE, A-domain	Rabbit	Synaptic Systems	192103	1:1000	AB_2086775
CtBP2 (RIBEYE)	Mouse	BD Biosciences	612044	1:1000	AB_399431
PSD-95	Mouse	Enzo Life Sciences	6G6-1C9	1:500	AB_10618933
Bassoon	Mouse	Enzo Life Sciences	ADI-VAM-PS003	1:400	AB_10618753
Dystrophin	Mouse	DSHB	MANDAG2(7D11)	1:50	AB_2211772
mGluR6	Sheep	*Cao et al.^51^	–	1:200	AB_2650490
TRPM1	Sheep	*Cao et al.^51^	–	1:1000	n/a
Mouse IgG: Alexa 488	Goat	Jackson ImmunoResearch	115-545-071	1:500	AB_2338847
Mouse IgG: Alexa 488	Goat	ThermoFisher	A-11001	1:500	AB_2534069
Rabbit IgG: Alexa 488	Goat	ThermoFisher	A-11008	1:500	AB_143165
Mouse IgG: Alexa 647	Goat	ThermoFisher	A-21235	1:500	AB_2535804
Rabbit IgG: Alexa 647	Goat	ThermoFisher	A-21244	1:500	AB_141663
Sheep IgG: Alexa 488	Donkey	ThermoFisher	A-11015	1:500	AB_2534082

* Kindly provided by Amy Lee of Ref. 32 and Kirill Martemyanov of Ref. 51.

### Image Analysis

Maximum through z-stack projections were used with manipulation of images limited to rotation, cropping, and adjusting the brightness and contrast levels using software (ImageJ, Zen Light 2009 [Carl Zeiss], or Adobe Photoshop CC [Adobe Systems, Inc., San Jose, CA, USA]). A minimum of two images per mouse for at least three mice per genotype per experiment were analyzed.

Ribbon length measurements were made by first outlining the border of electroporated presynaptic terminals in the OPL using mKate 2 or iSYP-RFP expression as a guide. A spline calibrated to the image scale bar was drawn through the center of the long axis of the RIBEYE-labeled ribbon, and the average length of two measurements was recorded (ImageJ). An average of 100 terminals from three to five individual mice was measured.

### Visually Guided Water Maze

Mice were trained to swim under ambient room lighting (luminance 11.1 cd/m^2^) in a 4-foot-diameter pool to a high-contrast visible escape platform as previously described.^44^ A series of 30 test trials over 6 days were conducted. After testing was completed, retina flat mounts from electroporated animals were collected to verify that the regions of retina expressing the electroporation/induction marker (mKate2 or iSYP-RFP) covered at least 10% of the retina. OCT imaging was used to select for animals with the least amount of retina damage. Briefly, mice were anesthetized with ketamine/xylazine, and tropicamide (1%) was used to dilate the pupils. Images were collected with a spectral-domain imaging system (Bioptigen, Inc., Morrisville, NC, USA) equipped with a mouse retina objective with the reference arm position set at 1264. Scan parameters were as follows: rectangular (1.4 mm^2^) volume scans, 1000 A-scans/B-scan, 33 B-scans/volume, 3 frames/B-scan, and 1 volume.

### Statistical Analysis

Statistical differences were determined using software (Prism, v. 8; GraphPad, San Diego, CA, USA). In the text, the mean is reported with the standard error of the mean (SEM), and in all graphs variability (SD) is shown. Mean ± SD is shown in all graphs. Statistical significance was defined using α = 0.05. Normality was assessed by the Shapiro-Wilks test; nonparametric data were analyzed using Mann-Whitney, and parametric data by *t*-test or ANOVA as indicated.

## Results

### Rescue Strategy

**Figure 1:**
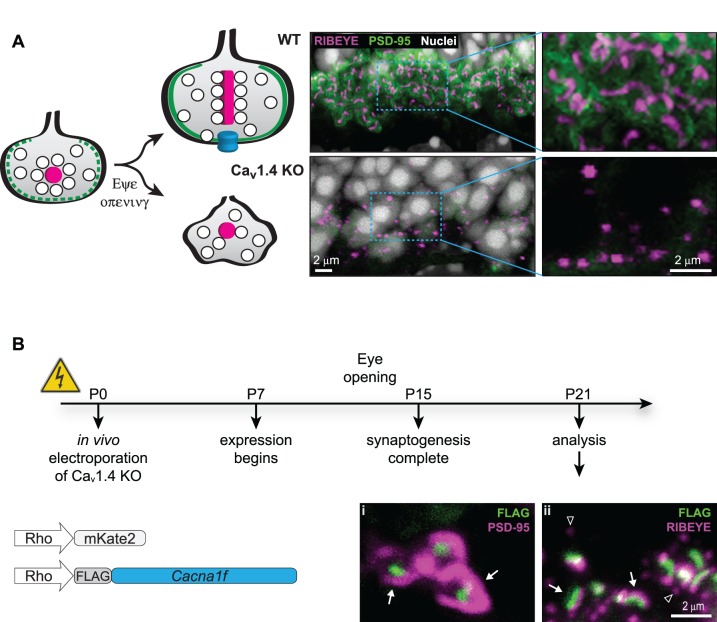
Synaptic development requires Ca_v_ α_1F_. (A) Left, schematic of the developing rod synaptic terminal lined with PSD-95 (green), containing synaptic vesicles and an immature, spherical ribbon (magenta). Coincident with eye opening, Ca_v_1.4 (cyan) becomes clustered beneath the mature, elongated ribbon. In the absence of Ca_v_1.4, the terminal fails to develop/degenerates instead of maturing. Right, outer plexiform layer labeled for RIBEYE (magenta) and PSD-95 (green) in WT (upper) or Ca_v_1.4 KO (lower) retina. Areas selected for high magnification indicated with blue dashed boxes. Nuclei were stained with Hoechst (white). (B) Experimental timeline with schematic of electroporated plasmids. High magnification images are labeled with FLAG (green) and PSD-95 (magenta, left) or RIBEYE (magenta, right). Arrows indicate rescued synapses, arrowheads mark immature spheres in KO synapses; all scale bars: 2 μm.

As shown previously,^31^–^34^,^42^,^52^ the lack of mature rod synapses in Ca_v_1.4 KO mice is reflected by the loss of PSD-95 and elongated ribbons in rod terminals (Fig. 1A). PSD-95 is a scaffolding protein lining the presynaptic membrane, and in Ca_v_1.4 KO retina it can be detected in the developing synapses before eye opening, but it relocalizes to the inner segment by 3 weeks of age.^42^ RIBEYE, the central component of the ribbon, was reduced in staining intensity and changed in shape from elongated ribbon to spherical. The spherical shape has been proposed to be a precursor form of the developing ribbon.^53^ Imaging of PSD-95 and RIBEYE were used throughout this study to assess the state of photoreceptor presynaptic development.

To enable exogenous expression of Ca_v_ α_1F_ in Ca_v_1.4 KO photoreceptors, we used in vivo electroporation to transfect rods. In this approach, pioneered by Cepko and colleagues,^48^,^49^ plasmid DNA is injected into the subretinal space of one neonatal eye and transcranial voltage pulses are applied to transfect rod precursors; cones are not transfected because they exit the cell cycle prenatally. In our experience, this approach resulted in sparse transfection of rods, at most 10% of rods within a transfected area of the retina that varied from 5% to 60%. The advantage of sparse transfection is the ability to compare treated and nontreated cells within the same image.

We electroporated FLAG-tagged mouse *Cacna1f* (coding for Ca_v_ α_1F_) and a fluorescent marker (mKate2, not shown in images for clarity) into Ca_v_1.4 KO retina. Expression of *Cacna1f* was under control of either the cytomegalovirus (CMV) ubiquitous promoter (data not shown) or the rod-specific rhodopsin promoter (Rho), both of which enable expression prior to rod synaptogenesis.^48^ Retinas were harvested and immunostained at P21, when photoreceptors are functionally mature despite ongoing growth of the outer segment.^54^,^55^ FLAG labeling of the presynaptic terminal coincided with expression of PSD-95 and RIBEYE-labeled ribbons, which were often elongated or arch-shaped, like a mature ribbon rather than the spherical form found in the adjacent FLAG-negative synaptic terminals (Fig. 1B). Additional markers for different subregions of the synapse were also restored (Supplemental Fig. S1). This demonstrates that expression of FLAG-Ca_v_ α_1F_ (hereafter referred to as Ca_v_ α_1F_) by in vivo electroporation is sufficient to support the morphologic development of the rod synaptic terminal.

**Figure 2:**
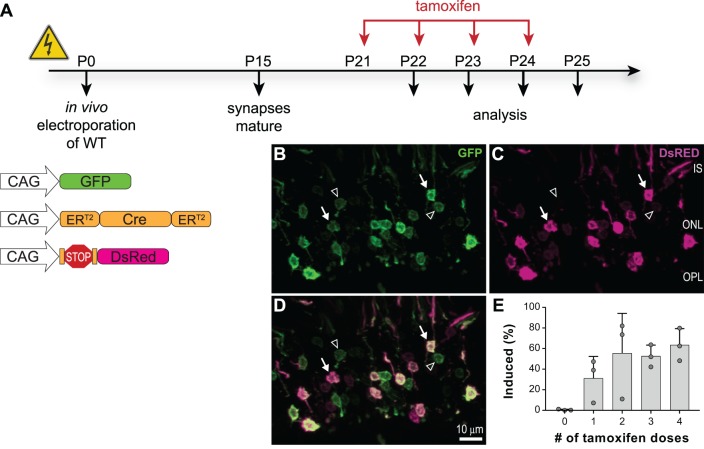
Validation of inducible gene-expression strategy. (A) Experimental timeline with schematic of electroporated plasmids. (B–D) Image of photoreceptors at P24 (after three doses of tamoxifen), GFP (green) marks electroporated cells, DsRed (magenta) marks electroporated and induced cells. Arrows mark example rod nuclei expressing both GFP and DsRed, arrowheads mark example rod nuclei expressing only GFP; scale bar: 10 μm. (E) Quantification of induction efficiency (proportion of DsRED to total GFP-positive nuclei); bars are mean + SD, and symbols are values from individual animals. IS, inner segment; ONL, outer nuclear layer; OPL, outer plexiform layer.

To achieve temporal control of Ca_v_ α_1F_ expression, we took advantage of a tamoxifen gene–induction strategy. This strategy consists of coelectroporation of the gene of interest preceded by a floxed stop codon and a tamoxifen-inducible version of Cre recombinase.^50^ We first performed a series of control experiments to determine the efficiency of gene induction using tamoxifen rather than the costlier 4-hydroxytamoxifen used in the original description of this method. WT mouse retinas were electroporated with green fluorescent protein (GFP) to mark electroporated cells, a Cre-controlled DsRED to report induced expression, and tamoxifen-inducible Cre recombinase (ER^T2^CreER^T2^). All plasmids contained the CAG promoter to drive constitutive expression. Beginning at P21, sequential doses of 1 mg tamoxifen were delivered by intraperitoneal injection every 24 hours for 4 days. Retinas were harvested after zero, one, two, three, or four doses of tamoxifen and induction efficiency determined by the ratio of cells expressing DsRed to cells expressing GFP (Fig. 2A). At least 50% of electroporated rods were induced with either two, three, or four doses of tamoxifen (Fig. 2B–D). We chose to use three doses of tamoxifen for all subsequent induction experiments.

### Morphology of Synaptic Terminals Rescued in Adulthood

**Figure 3:**
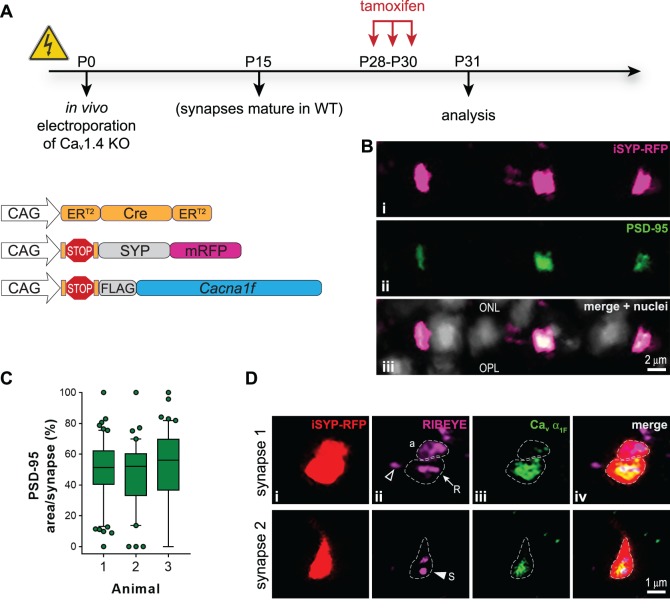
Brief Ca_v_ α_1F_ expression in mature retina rescues rod synapse morphology. (A) Experimental timeline with schematic of electroporated plasmids. (B) PSD-95 labeling in the OPL of electroporated and induced Ca_v_ KO retina; (i) iSYP-RFP (magenta), (ii) PSD-95 (green), and (iii) merged image with Hoechst-labeled nuclei (white). (C) Quantification of PSD-95 amount per terminal; box and 5% to 95% whiskers; individual synapses outside that range are shown as symbols. (D) RIBEYE and Ca_v_ α_1F_ labeling of electroporated and induced Ca_v_1.4 KO retina synapses; (i) iSYP-RFP (red), (ii) RIBEYE (magenta), (iii) Ca_v_ α_1F_ (green), and (iv) merged image. Three patterns of RIBEYE labeling were observed; amorphous (a), elongated ribbon (arrow, R) in induced synapses, or spherical (S) in both induced (closed triangle) and KO synapses (open triangle). Scale bars: 2 μm (B) and 1 μm (D).

With the induction strategy verified, plasmids for an inducible version of Ca_v_ α_1F_ (hereafter referred to as i-α_1F_), along with an inducible fluorescent marker for synaptic vesicles, synaptophysin-mRFP (iSYP-RFP), and ER^T2^CreER^T2^ were electroporated into Ca_v_1.4 KO retina. Tamoxifen was delivered on P28, P29, and P30, and retinas were harvested on P31 (Fig. 3A). PSD-95 labeling was observed in almost all rods expressing the iSYP-RFP marker (Fig. 3B). We examined an average of 104 iSYP-RFP expressing rod terminals from each of three different mice and found that 97% of the iSYP-RFP terminals expressed PSD-95. The amount of PSD-95, which we recorded as the area of PSD-95 label normalized to the area of iSYP-RFP label per terminal, ranged dramatically from the few terminals with no PSD-95 to some being completely filled. The average area of the terminal filled with PSD-95 was 51.5% ± 1.7% (Fig. 3C). We conclude that induction of Ca_v_ α_1F_ rescues PSD-95 expression in the adult retina.

Ribbon morphology in Ca_v_ α_1F_-induced terminals was variable, taking on one of three major shapes: amorphous, elongated/arched ribbon (elongated RIBEYE labeling with the horizontal axis at least twice as long as the vertical axis), or spherical (circular RIBEYE labeling with horizontal and vertical axis shorter than 1 μm) (Fig. 3D). We expected that Ca_v_ α_1F_ would localize in a pattern mirroring that of the ribbon as seen when Ca_v_ α_1F_ expression began before eye opening (Fig. 1Bii), but instead we found Ca_v_ α_1F_ labeling was amorphous in the center of the terminal independent of the shape of the ribbon. To follow up on this observation we repeated the experiment but allowed for more time between inducing Ca_v_ α_1F_ and the analysis—from an approximately 1-day to approximately 3-week interval; these two experiments are hereafter referred to as i-α_1F_ (1 day) versus i-α_1F_ (3 week).

**Figure 4:**
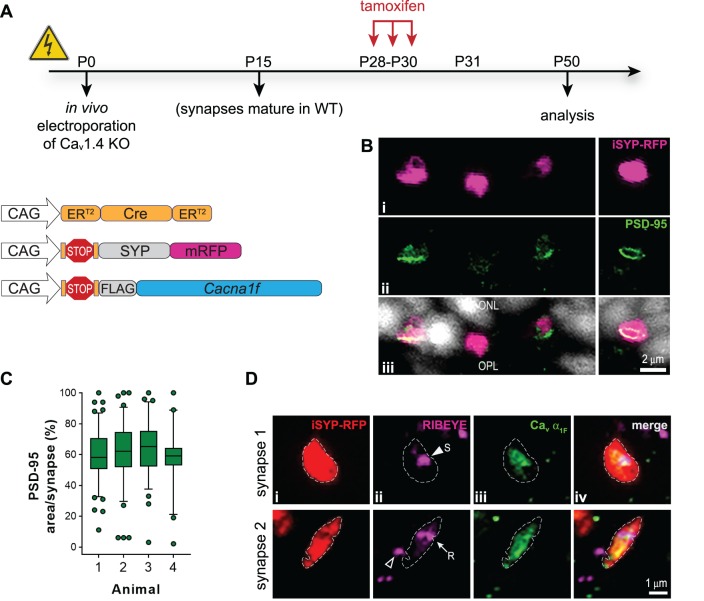
Prolonged Ca_v_ α_1F_ expression in mature retina rescues rod synapse morphology. (A) Experimental timeline with schematic of electroporated plasmids. (B) PSD-95 labeling in the OPL of electroporated and induced Ca_v_1.4 KO retina: (i) inducible SYP-RFP (magenta), (ii) PSD-95 (green), and (iii) merged image with Hoechst-labeled nuclei (white). (C) Quantification of PSD-95 amount per terminal: box and 5% to 95% whiskers, individual synapses outside that range are shown as symbols. (D) RIBEYE and Ca_v_ α_1F_ labeling of electroporated and induced Ca_v_1.4 KO retina synapses: (i) inducible SYP-RFP (red), (ii) RIBEYE (magenta), (iii) Ca_v_1.4 α_1F_ (green), and (iv) merged image. Scale bars: 2 μm (B) and 1 μm (D).

In the prolonged interval, i-α_1F_ (3-week) experiment, tamoxifen was delivered on P28, P29, and P30, and retinas were harvested on P50 (Fig. 4A). As in the i-α_1F_ (1-day) experiment, PSD-95 labeling was detected in 97% of the induced terminals; an average of 84 iSYP-RFP expressing rod terminals from each of four different mice were examined (Fig. 4B). The amount of PSD-95 in the terminal again exhibited the full range, and the average area of the terminal filled was 62.9% ± 1.3%; the increased value from i-α_1F_ (1 day) to i-α_1F_ (3 weeks) was not statistically significant (Δ 12.8%; Mann-Whitney, *P* = 0.06) (Fig. 4C). Ribbon morphology was variable, and Ca_v_1.4 labeling was again amorphous (Fig. 4D). This experiment demonstrates that the length of time Ca_v_1.4 α_1F_ is expressed in a mature KO rod does not change the degree of morphologic rescue. The amorphous nature of Ca_v_ α_1F_ labeling could reflect excessive expression compared to the expression levels in WT rods, an imbalance in the expression levels of α_1F_ and the accessory subunits, or simply be an indication of some unmeasured abnormality in the older Ca_v_1.4 KO terminals.

**Figure 5:**
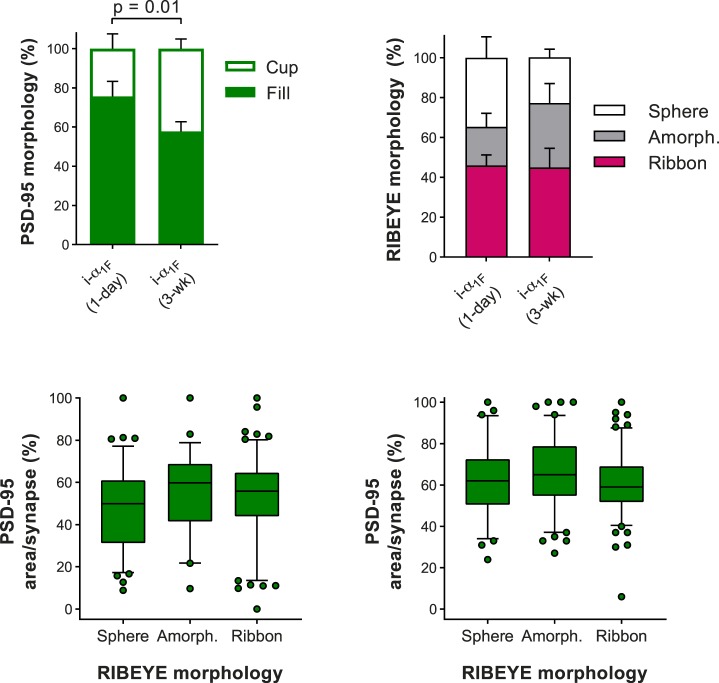
Comparison of synaptic features analyzed at different times post induction of Cav α_1F_ in adulthood. (A) Comparison of terminals with cup-like or filled PSD-95 morphology and (B) RIBEYE morphology defined as a sphere, amorphous, or a ribbon. (C, D) Quantification of PSD-95 amount in terminals containing either RIBEYE in a sphere, amorphous, or a ribbon; box and 5% to 95% whiskers, with individual synapses outside that range shown as symbols for (C) i-α_1F_ (1 day) or (D) i-α_1F_ (3 week).

We further analyzed the larger dataset of synapses (>300 each) labeled with PSD-95 and RIBEYE to identify differences between the i-α_1F_ (1-day) and i-α_1F_ (3-week) experiments. The morphology of PSD-95 in WT rod terminals lines the plasma membrane so that the labeling looks cup-like. In the rescue experiments, PSD-95 labeling most often filled the terminal but did sometimes appear cup-like: 24% ± 4% versus 42% ± 2% in the i-α_1F_ (1-day) versus i-α_1F_ (3-week) experiments, which was a statistically significant increase (Δ18%, 95% CI [6, 30] *t*-test, *P* = 0.01) (Fig. 5A). The morphology of RIBEYE was similar between i-α_1F_ (1-day) versus i-α_1F_ (3-week) experiments: 46% ± 3% and 42% ± 4% of terminals contained spherical, 31% ± 6% and 21% ± 2% amorphous, or 19% ± 4% and 30% ± 5% ribbon-shaped RIBEYE, respectively. These minor differences were not statistically significant (*t*-test, *P* = 0.12, 0.16, or 0.42, respectively) (Fig. 5B). There was no correlation between the morphology of RIBEYE and the amount of PSD-95 in the terminal for either experiment, demonstrating that these are independent measures (Fig. 5C, D). In summary, allowing more time for Ca_v_ α_1F_ to be expressed in the terminal is not necessary to restore either robust PSD-95 expression or elongated ribbons. More surprisingly, we continued to observe Ca_v_ α_1F_ diffusely labeling the terminal independent of ribbon shape, indicating that Ca_v_ α_1F_ just needed to be in the terminal in order to stabilize PSD-95 expression and support ribbon elongation.

**Figure 6:**
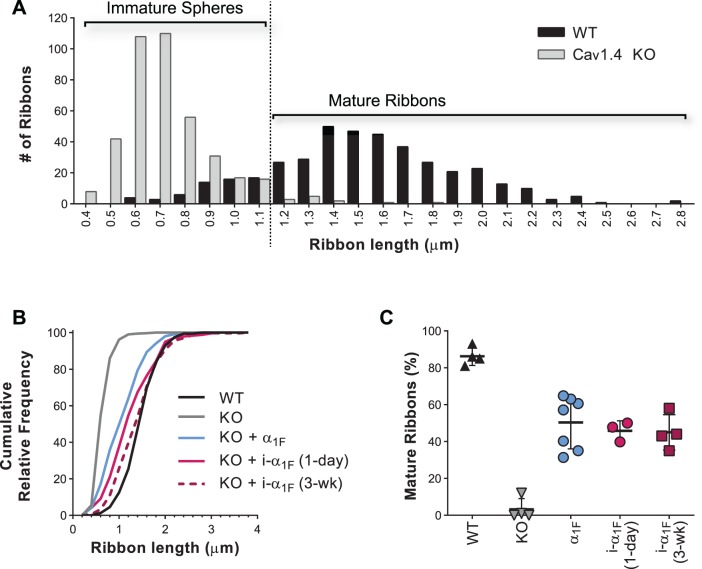
Expression of Ca_v_ α_1F_ restores ribbon elongation either before or after eye opening. (A) Histogram of rod ribbon lengths in WT (black) or Ca_v_1.4 KO to illustrate the designation of a RIBEYE-labeled object as an immature sphere or mature ribbon. (B) Cumulative frequency plots of rod ribbon lengths in WT (black), KO (gray), KO + Ca_v_ α_1F_ (KO + α_1F_, cyan), KO + inducible Ca_v_ α_1F_ (i-α_1F_) analyzed at 1 day post induction (magenta), or KO + i-α_1F_ analyzed at 3 weeks post induction (dark magenta, dashed). (C) Proportion of mature ribbons in rod synapses from WT (black), KO (gray), KO + α_1F_ (cyan), KO + i-α_1F_ analyzed at 1 day (magenta) or 3 weeks (dark magenta) post induction. Lines are mean ± SD and symbols are average values from individual animals.

Ribbon length in Ca_v_ α_1F_-induced terminals was measured to provide another metric of presynaptic rescue. To establish a baseline we measured 100 ribbons from each of four WT and four Ca_v_1.4 KO animals. The lengths of WT rod ribbons ranged from 0.6 to 2.8 μm, with a mean of 1.5 μm. This agrees with previous reports, and the fairly large range is likely due to a combination of the dynamic nature of ribbons and sectioning plane, since an elongated ribbon sliced en face appears spherical. In Ca_v_1.4 KO rods, RIBEYE was found in immature spheres with a mean diameter of 0.7 μm (Fig. 6A). The difference in these two ribbon populations is easier to visualize in cumulative frequency plots where the WT ribbons are shifted toward longer lengths and the distribution has a shallower slope than the KO “ribbons” (Hill slope of 1.98 versus 4.44, respectively; Fig. 6B). Next, we compared the ribbon lengths in electroporated KO terminals from the experiments described in Figures 1, 3, and 4 (for amorphous or spherical RIBEYE-labeled structures, we measured the average diameter). Sigmoidal fits of the cumulative frequency plot highlight that the ribbons in the treated Ca_v_1.4 KO were significantly different from untreated Ca_v_1.4 KO (Table 3).

**Table 3:** Ribbon Length

**Genotype**	**Mean Length**	**Hill Slope**	**Hill Slope, Differences From KO**	**ANOVA + Dunnett's, Adjusted** ***P*** **Value**
KO	0.7 μm	4.44		
KO + Ca_v_ α_1F_	1.1 μm	1.38	Δ3.06, 95% CI [2.53, 3.59]	<0.0001
KO + i-α_1F_ (1 d)	1.3 μm	1.28	Δ3.16, 95% CI [2.63, 3.69]	<0.0001
KO + i-α_1F_ (3 wk)	1.3 μm	1.39	Δ3.05, 95% CI [2.52, 3.58]	<0.0001

We also considered a simpler analysis of the ribbon length where we binned ribbons into immature or mature based on the criterion that a mature ribbon is elongated. Using the mean rod ribbon length in WT minus 1 SD as the cutoff; ribbons in electroporated rods >1.13 μm were scored as mature (Fig. 6A). Then we compared the proportion of terminals containing mature ribbons per animal across experiments. Rescue by this metric in animals expressing Ca_v_ α_1F_ prior to eye opening (α_1F_), or post eye opening i-α_1F_ (1 day), or i-α_1F_ (3 week) was 50.3% ± 5.4%, 48.7% ± 5.8%, or 58.4% ± 2.8%, which was not significantly different from each other (ANOVA, *P* = 0.48). Note that the proportion of mature ribbons in WT animals was less than 100% (86.3% ± 2.5%) because ribbons are oriented in different planes and ribbons cut at an angle or en face appear short or spherical. In turn, there were some ribbons scored as mature in the KO (3.3% ± 2.9%), likely because not all adjacent spheres were spatially resolved (Fig. 6D). From this simplified analysis of ribbon length, we conclude that ribbon elongation can be rescued to the same extent when Ca_v_ α_1F_ is introduced before or after eye opening, that is, before or after rod synaptogenesis is normally complete.

### Vision-Guided Behavior of Treated Animals

**Figure 7:**
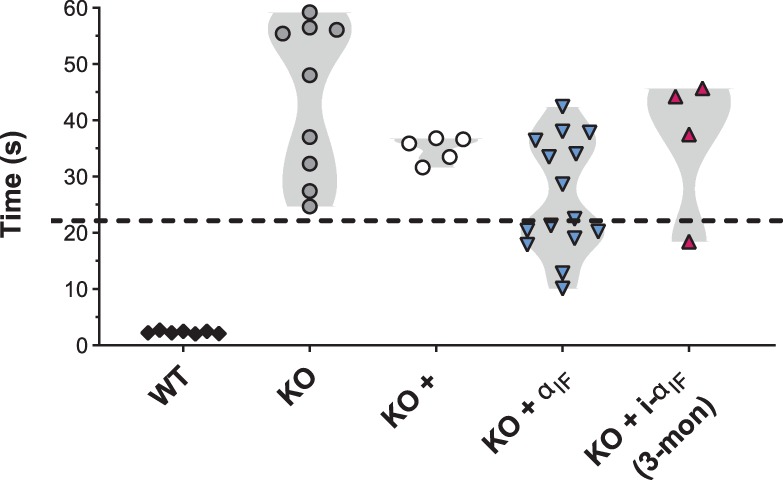
Limited performance improvement in a visually guided water maze. The average time to swim to a high-contrast visible escape platform for individual animals. Shading highlights the frequency distribution for the various groups: (1) WT, (2) Ca_v_1.4 KO (KO), (3) Ca_v_1.4 KO electroporated with the marker mKate2 (KO +), (4) Ca_v_1.4 KO electroporated with mKate2 and Ca_v_ α_1F_ (KO + α_1F_), or (5) Ca_v_1.4 KO electroporated with ER^T2^CreER^T2^, iSYP-RFP, and i-α_1F_, induced at 1 month of age, tested 3 months later (KO + i-α_1F_ (3-mon)). All animals were between 2 and 4 months of age at time of testing. The dashed line at 22 seconds is just below the lower limit for the performance of Ca_v_1.4 KO control animals.

We used a water maze to determine if the morphologically restored rod ribbon synapses were capable of supporting vision. In this task, mice were trained to swim in a pool to a randomly placed visible escape platform, then the average swim duration for 30 test trials conducted over 6 days is recorded. Short swim latencies reflect intact visual function.^56^–^58^ WT mice completed the task with a group average of 2.3 ± 0.1 seconds as they swam directly to the escape platform. Ca_v_1.4 KO mice wandered around the pool, taking an average of 44.1 ± 4.6 seconds (Fig. 7; Table 4).^44^ We tested a cohort of Ca_v_1.4 KO animals electroporated with the mKate2 marker alone to make sure the electroporation itself did not change the behavior of the animals. As expected, none of those mice passed the water maze (∼35-second swim latency).

**Table 4:** Statistical Description of Water Maze Data

**Genotype**	***n***	**Mean ± SEM**	**Skewness**	**Mean Differences Compared to KO**	**ANOVA + Dunnett's, Adjusted** ***P*** **Value**
WT	7	2.3 ± 0.1 s	0.3		
KO	9	44.1 ± 4.6 s	−0.3		
KO + marker	5	34.9 ± 1 s	−0.9	Δ9.2 s, 95% CI [−5.1, 23.4]	0.3095
KO + Ca_v_ α_1F_	15	26.3 ± 2.6 s	0.1	Δ17.7 s, 95% CI [6.9, 28.5]	0.0006
KO + i-α_1F_ (3-mon)	4	36.5 ± 6.3 s	−1.6	Δ7.6 s, 95% CI [−7.7, 23.0]	0.5406

The cohort of Ca_v_1.4 KO animals electroporated with constitutively expressed Ca_v_ α_1F_ (see Fig. 1) had an average swim latency of 26.3 ± 2.6 seconds, which was a significant improvement compared to the untreated KO (Table 4). We next electroporated Ca_v_1.4 KO animals with i-α_1F_, treated with tamoxifen from P28 to P30, then used OCT imaging (data not shown) to screen for gross retinal detachments between 2 and 3 months of age. The majority (23 out of 27) of animals had large retinal detachments that precluded water maze testing. Of the remaining four animals, which were tested at 4 months of age (3 months post Ca_v_1.4 α_1F_ induction), the average swim latency was 36.5 ± 6.3 seconds, which was not different from the negative control.

However, that data had a large degree of asymmetrical distribution (skewness > 1) due to the performance of one induced animal with a latency of 18.4 ± 2.9 seconds (Fig. 7). Despite the sample size for this experiment being so drastically limited by persistent retinal detachments, the evidence for vision sufficient to navigate the water maze in one animal from the induced KO cohort is remarkable. Consider that performance is not likely to reach the range of WT animals because electroporated animals are treated in only one eye, and the number of electroporated rods across the retina varies markedly between animals but is usually quite low. While Ca_v_1.4 KO mice exhibit a range of swim latencies (Fig. 7); the best performing Ca_v_1.4 KO animal had a latency of 27.4 ± 3.2 seconds. If we set an arbitrary cutoff just below that minimum, at <22 seconds, as passing the water maze for individual electroporated animals, then half of the animals (7 out of 15) electroporated for constitutive expression of Ca_v_ α_1F_ exhibited visually guided behavior. In the case of the animals electroporated for induction of Ca_v_ α_1F_ expression in maturity, the probability of restoring sufficient vision to pass this test is further reduced since only half of the electroporated cells were likely induced (see Fig. 2). Using the water maze test as a proof-of-principle type experiment, we conclude that the synaptic rescue scored by morphologic criteria corresponds to functionally restored rod terminals that can support vision.

## Discussion

The key finding of this study is that rod synaptic terminals that failed to develop due to loss of Ca_v_1.4 can be restored in both immature and mature retinas. The ability to rescue the loss of Ca_v_ α_1F_ with exogenous Ca_v_ α_1F_ in immature retinas is not surprising, but it confirms that Ca_v_ α_1F_ is necessary for maturation of the rod synaptic terminal. The ability of exogenous Ca_v_ α_1F_ to rescue multiple features of synaptic maturation in mature retinas was more surprising, especially since all of our quantitative metrics demonstrated that rescue was as effective when it occurred either before or after eye opening. This is noteworthy because in the mature animals the rod synaptic terminals are so malformed that they are largely unrecognizable by electron microscopy.^31^,^52^ These findings indicate the rod synaptic terminal maintains substantial regenerative capacity—an observation that provides added optimism for the success of future gene or cell replacement therapies for IRDs.

The synaptic plasticity observed in this study is consistent with previous findings regarding the dynamic nature of the ribbon. In addition to the remodeling that occurs from disease or as a part of aging, there can be environmentally regulated changes in the synaptic ribbon that seem to benefit the animal. In the albino Balb/c mouse strain, the ribbon disassembles rapidly in response to light, which is likely to be a protective adaptation to excessive light exposure (this does not occur in the pigmented C57Bl/6 strain used in this study).^59^ In the cone-rich retina of ground squirrels, which undergo seasonal hibernation, there is a rapidly reversible loss of synaptic vesicles and ribbons from cone terminals of animals undergoing torpor.^60^ This is accompanied by a reduction in synaptic vesicle release, which is a major energy-consuming process, and therefore likely to be of benefit in helping the animal conserve precious resources.^61^,^62^ A study similar in concept to the present one—asking if treatment of adult retinas would be too late—found that rescue of the essential phototransduction effector enzyme, PDE6, in a retinitis pigmentosa mouse model halted disease progression at all stages that were tested. In that study, the morphology of rod synapses were not directly examined, but the photopic electroretinogram (ERG) b wave that reflects transmission across the first visual synapse was rescued.^63^

As with any study design there are technical caveats that should be considered. In our opinion, the major limitation to the approach of photoreceptor in vivo electroporation is the high probability of causing retinal damage, that is, formation of neural rosettes or retinal detachment. Detachment is a necessary part of any subretinal injection, and in this case, it occurs on the day of birth, approximately 10 days before the photoreceptors develop outer segments that interdigitate with microvilli from RPE cells, an interaction that would greatly facilitate resolution of the detachment. We think this issue had the largest negative impact by limiting the number of animals that could be tested in the behavioral assay.

One of the diagnostic features of Ca_v_1.4 loss of function is an electronegative b wave in ERGs. We made several attempts to record ERGs from electroporated Ca_v_1.4 KO animals and found no differences compared to the recordings from nonelectroporated animals. There are technical issues that could explain those negative results. First, the retinal damage discussed above would negatively impact the ERG since the waveforms are the summed potential of the entire retina. Second, the low efficiency of the electroporation procedure could be below the threshold for the number of functional photoreceptor-to-rod ON bipolar synapses required to generate the typical b wave. Finally, the lack of a restored ERG does not negate the water maze test because the electronegative b wave does not a priori mean the animals lack vision.^44^,^64^–^66^

Another limitation is due to the efficiency of in vivo electroporation, both in the absolute number of cells transfected and in the variable expression levels from the plasmids. The variable expression levels can be readily seen in the GFP control (Fig. 2) and was likely the driving factor for the large range of PSD-95 expression and ribbon lengths that we observed. If more rods could be rescued, then perhaps the performance of treated Ca_v_1.4 KO animals would more closely approach that of the WT animals. On the other hand, the sparse transfection is a great benefit when it comes to being able to clearly distinguish treated from nontreated rod synaptic terminals in our imaging studies.

Our approach does not allow us to determine if cones also maintain regenerative capacity. Cone synaptic terminals are structurally and functionally distinct from those of rods.^67^ They develop earlier and form conventional flat synapses as well as numerous invaginating ribbon synapses that communicate with an array of cone bipolar cell types. Mutations in Ca_v_1.4 subunits do not always affect cone synapse morphology as severely as it does rods.^31^,^36^,^44^–^47^,^52^ If the factor(s) that support cone ribbon development in the absence of Ca_v_1.4 could be identified, it would be interesting to test if that factor could further enhance the plasticity of rod synapses.

The challenge for future studies is to determine the mechanism by which Ca_v_1.4 triggers synaptogenesis. It may be a Ca^2+^-dependent signaling event that acts locally or ultimately affects transcription.^68^,^69^ Alternatively, Ca_v_1.4 could play a structural role in organizing the synaptic terminal. Signaling proteins often have multiple functions, with rhodopsin being the prime example of a photoreceptor protein taking on both signaling and structural roles.^70^ If the mechanism of Ca_v_1.4-mediated synaptogenesis was deciphered, then it could inform the development of approaches to boost photoreceptor synaptic development that could conceivably be used to either extend the functional lifetime of diseased rods or increase the integration of transplanted cells.

## Supplementary Material

Supplement 1

Supplement 2
