# Supplementary material for: Rescue of Rod Synapses by Induction of Cav Alpha 1F in the Mature Cav1.4 Knock-Out Mouse Retina
Source: Invest Ophthalmol Vis Sci. 2019 Jul;60(8):3150–61. doi: 10.1167/iovs.19-27226 (PMC6656410; doi:10.1167/iovs.19-27226)
Supplement: Supplement 1 [file iovs-60-08-23_s01.pdf]

Supplemental Figure 1 (related to Fig 1B)

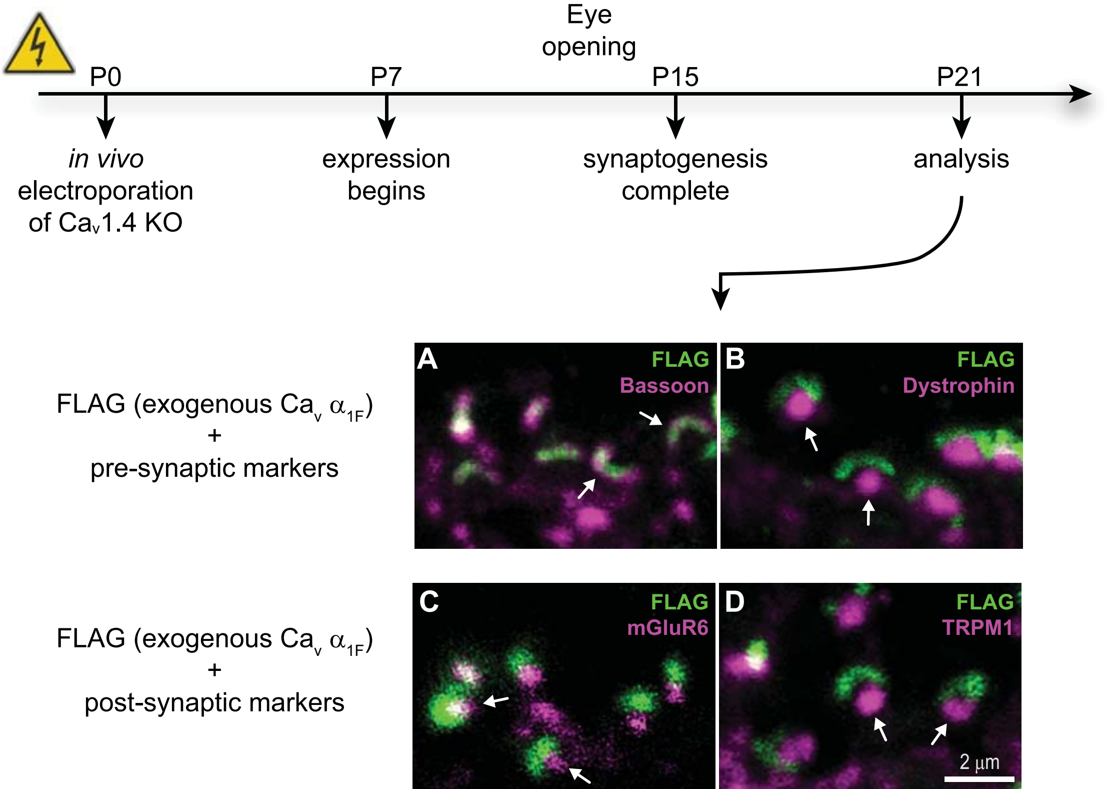

Supplemental Figure 1: *Exogenous expression of Ca_v α_{1F} rescues both pre- and post-synaptic markers.* Retinal sections from the experiment described in Figure 1B were co-labeled with FLAG (green) to detect the exogenous Ca_v α_{1F} along with additional synaptic markers (magenta): ***A)*** Bassoon, ***B)*** Dystrophin, ***C)*** mGluR6, ***D)*** TRPM1. Arrows indicate example rescued synapses; all scale bars, 2 μm.
