# Supplementary figures and images for: Rescue of Rod Synapses by Induction of Cav Alpha 1F in the Mature Cav1.4 Knock-Out Mouse Retina
Source: Invest Ophthalmol Vis Sci. 2019 Jul;60(8):3150–61. doi: 10.1167/iovs.19-27226 (PMC6656410; doi:10.1167/iovs.19-27226)

# Graphical Abstract

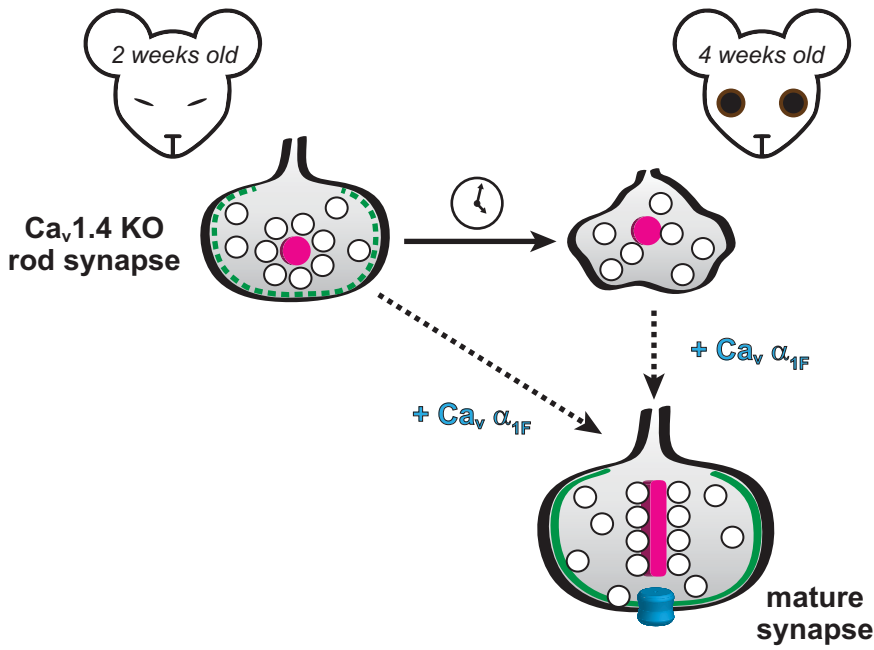

Supplement: Supplement 2 [file iovs-60-08-23_s02.pdf]
